# ATP/ADP biosensor organoids for drug nephrotoxicity assessment

**DOI:** 10.3389/fcell.2023.1138504

**Published:** 2023-03-02

**Authors:** Koichiro Susa, Kenichi Kobayashi, Pierre Galichon, Takuya Matsumoto, Akitoshi Tamura, Ken Hiratsuka, Navin R. Gupta, Iman K. Yazdi, Joseph V. Bonventre, Ryuji Morizane

**Affiliations:** ^1^ Renal Division, Department of Medicine, Brigham and Women’s Hospital, Boston, MA, United States; ^2^ Harvard Medical School, Boston, MA, United States; ^3^ Department of Nephrology, Tokyo Medical and Dental University, Tokyo, Japan; ^4^ Massachusetts General Hospital, Boston, MA, United States; ^5^ Wyss Institute for Biologically Inspired Engineering, Harvard University, Boston, MA, United States; ^6^ Division of Engineering in Medicine, Department of Medicine, Brigham and Women’s Hospital, Boston, MA, United States; ^7^ Harvard-MIT Division of Health Sciences &Technology, Massachusetts Institute of Technology, Cambridge, MA, United States; ^8^ Harvard Stem Cell Institute, Cambridge, MA, United States

**Keywords:** nephron, organoid, ATP, drug development, transporter, kidney, KIM-1

## Abstract

Drug nephrotoxicity is a common healthcare problem in hospitalized patients and a major limitation during drug development. Multi-segmented kidney organoids derived from human pluripotent stem cells may complement traditional cell culture and animal experiments for nephrotoxicity assessment. Here we evaluate the capability of kidney organoids to investigate drug toxicity *in vitro*. Kidney organoids express renal drug transporters, OAT1, OAT3, and OCT2, while a human proximal tubular cell line shows the absence of OAT1 and OAT3. Tenofovir and aristolochic acid (AA) induce proximal tubular injury in organoids which is ameliorated by an OAT inhibitor, probenecid, without damage to podocytes. Similarly, cisplatin causes proximal tubular damage that can be relieved by an OCT inhibitor, cimetidine, collectively suggesting the presence of functional OATs and OCTs in organoid proximal tubules. Puromycin aminonucleoside (PAN) induced segment-specific injury in glomerular podocytes in kidney organoids in the absence of tubular injury. Reporter organoids were generated with an ATP/ADP biosensor, which may be applicable to high-throughput screening in the future. In conclusion, the kidney organoid is a useful tool for toxicity assessment in the multicellular context and may contribute to nephrotoxicity assessment during drug development.

## Introduction

Recently, several *in vitro* protocols involving either directed differentiation or transcription factor-based reprogramming into kidney cells and organoids have been established. These biotechnological advancements have enabled the generation of nephron progenitor cells and kidney organoids from human pluripotent stem cells (hPSCs) including embryonic stem cells (ESCs) and induced pluripotent stem cells (iPSCs) (Mae et al., 2013; Taguchi et al., 2014; Morizane et al., 2015; Takasato et al., 2016; Morizane and Bonventre, 2017b; Gupta et al., 2017; Morizane et al., 2017). Kidney organoids contain epithelial nephron-like structures expressing markers of podocytes, proximal tubules, loops of Henle, and distal nephrons in an organized, continuous arrangement that resembles the nephron *in vivo* (Morizane et al., 2015). There are various reports in which kidney organoids have been used for the investigation of inherited kidney diseases such as polycystic kidney disease (Cruz et al., 2017; Hiratsuka et al., 2022), acute kidney injury (AKI), and fibrosis (Lemos et al., 2018; Gupta et al., 2022), whereas it is controversial how well the present kidney organoids recapitulate human kidney pathophysiology for drug toxicity assessment.

Drug nephrotoxicity leads to the onset or aggravation of acute kidney injury in patients. Drug-induced kidney injury accounts for 7% of all drug toxicities, and the kidney is considered an organ susceptible to drugs and toxicants because of high blood flow and active drug transport in renal tubular epithelia. Even though the two kidneys account for only 0.4% of the total body weight, the kidneys receive 25% of cardiac output, resulting in high exposure to administered drugs. In addition, the glomerular filtrate is concentrated in tubular lumens due to renal water reabsorption, leading to tubular exposure to high concentrations of drugs. Drugs are not only filtered by glomeruli but also excreted by tubules *via* transporters such as organic anion transporters (OATs) or organic cation transporters (OCTs), increasing drug exposure to tubular cells (Nigam et al., 2015).

Drug-induced nephrotoxicity is one of the major healthcare problems, that causes acute kidney injury (AKI) and exacerbates chronic kidney disease (CKD). Cohort studies of AKI report the incidence of drug-induced nephrotoxicity is approximately 10%–33% in adult populations who receive medications including aminoglycosides, radiocontrast, antibiotics, antiretroviral drugs, statins, proton pump inhibitors, non-steroidal anti-inflammatory drugs, fibrates, and calcineurin inhibitors (Mehta et al., 2004; Rewa and Bagshaw, 2014). Hospitalized patients, particularly in ICUs, often need to take multiple drugs that can cause nephrotoxicity. In the ICU setting, the incidence of drug-induced AKI ranges between 1%–23% (Brivet et al., 1996; Liaño et al., 1998; Silvester et al., 2001; Mehta et al., 2004). Antimicrobials and contrast media are common causes of nephrotoxicity in hospitalized patients. Drug-induced nephrotoxicity is also a major barrier to drug discovery. While nephrotoxicity is detected in only 7% of new drug candidates in preclinical trials, more than 30% of new drugs tested in clinical trials are terminated because of adverse effects (Fuchs and Hewitt, 2011; Sun et al., 2022). These data indicate the prediction of nephrotoxicity is difficult with currently available preclinical tools during drug development, and novel toxicity assays are necessary to address the issue of clinical trial failure and enormous costs for drug development.

In this study, we evaluate the capability of kidney organoids to assess nephrotoxicity caused by various drugs in different nephron segments of proximal tubules and podocytes. Drug transporter expression and injury responses are assessed in comparison to a human proximal tubular cell line, a commonly used tool for nephrotoxicity assessment. In addition, we generated a reporter system using a biosensor of ATP and ADP in kidney organoids, which enabled real-time toxicity monitoring.

## Results

### hPSC-derived kidney organoids express organic anion/cation transporters in proximal tubules

**FIGURE 1:**
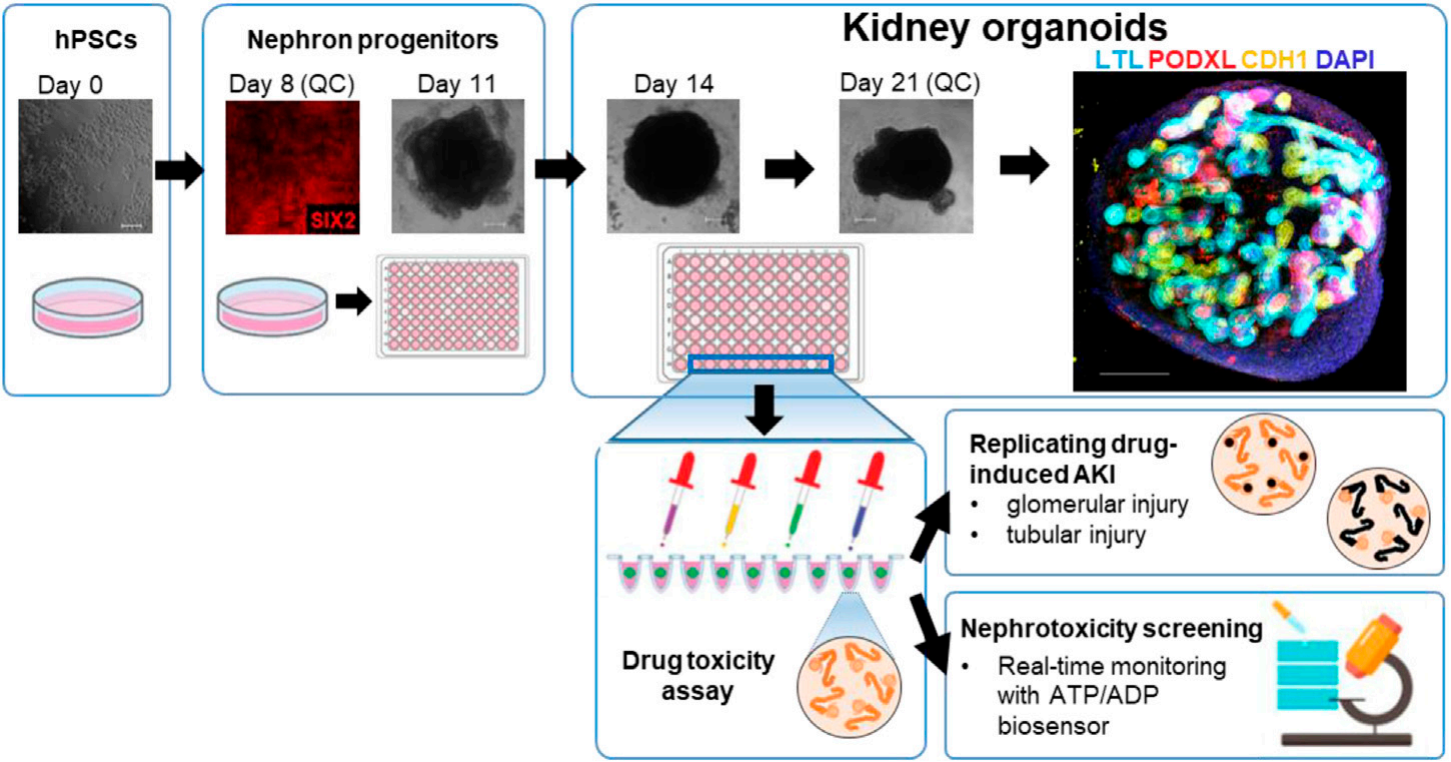
Representative steps of the generation of kidney organoids from hPSCs for the nephrotoxicity assays.

Two hPSCs lines, H9 ES cells and iPSCs from a healthy human, were used for differentiation into kidney organoids. The healthy iPSC line (TCiPS1) was reprogrammed from T lymphocytes obtained from a subject with no known pre-existing disease. Isolated blood T lymphocytes were expanded on tissue culture plates coated with anti-CD3 antibodies and reprogrammed by Sendai virus vectors with 4 transcription factors of *OCT3/4*, *SOX2*, *KLF4,* and *c-MYC*. Approximately 20 days after the transduction of Sendai virus, iPSC colonies were expanded and maintained on Geltrex-coated plates without feeder cells (Supplementary Figure S1). Kidney organoids were differentiated from H9 and TCiPS1 cells using a previously published protocol whereby metanephric kidney development was simulated by stepwise treatment with combinations of growth factors (Morizane et al., 2015; Morizane and Bonventre, 2017a). During the differentiation, quality control was performed at 2 different stages to minimize the batch variations of experiments. Differentiated cells on day 8 were evaluated for the expression of SIX2, a marker of nephron progenitor cells, and subsequent nephron formation was confirmed by immunostaining for PODXL (podocytes), lotus tetragonolobus lectin (LTL, proximal tubules), and CDH1 (loops of Henle/distal nephrons) (Figure 1).

Nephrotoxicity is one major side effect of clinically used drugs, and their cellular uptake is mediated by organic anion and cation transporters (Soo et al., 2018). The family of organic anion transporters (OATs) is a group of multispecific membrane transport proteins that have broad substrate preferences. OATs play a central role in the handling of negatively charged drugs (e.g., non-steroidal anti-inflammatory drugs, diuretics, and antibiotics), environmental toxins (e.g., ochratoxin A and mercuric chlorides), and other organic compounds (e.g., steroids, odorants, cyclic nucleotides, and neurotransmitters) (Eraly et al., 2004; Kaler et al., 2006; Truong et al., 2008). OAT1 and OAT3 are expressed in S1-S3 segments of proximal tubules on basolateral membranes and are responsible for organic anion secretion whereby drugs are concentrated in tubular cell cytoplasm (VanWert et al., 2010; Breljak et al., 2016). Organic cation transporter 2 (OCT2) is another drug transporter expressed in proximal tubules (Motohashi et al., 2013), and is involved in the uptake of positively charged drugs (e.g., metformin, cisplatin, and antihistamines), uremic metabolites (e.g., creatinine), toxins (e.g., ethidium), and other organic compounds (Pietig et al., 2001; Ciarimboli et al., 2005; Nigam et al., 2015). Podocytes also express another OCT, namely, plasma membrane monoamine transporter (PMAT), which is a member of the equilibrative nucleoside transporter (ENT) family that is predominantly localized in podocytes, which transports organic cations and the purine nucleoside adenosine by a sodium-independent and pH-sensitive mechanism (Zennaro et al., 2014).

**FIGURE 2:**
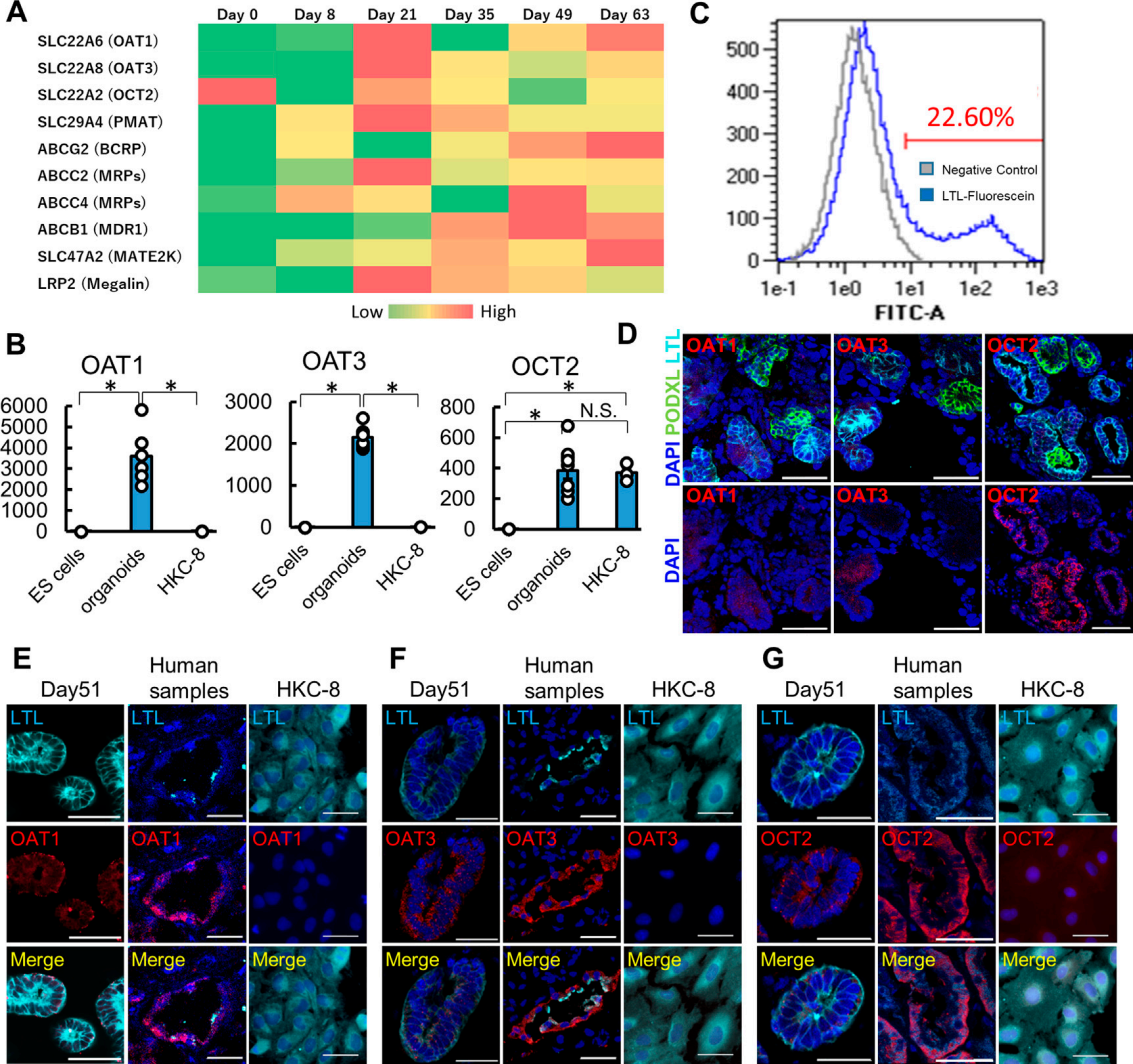
Maturation-dependent expression of renal drug transporters in kidney organoids, and comparison with human kidneys and HKC-8 cultured cells. **(A)** Heatmap for RNA-seq of drug transporters icluding OAT1, OAT3, OCT2, and PMAT in undifferentiated ES cells (day 0), nephron progenitor cells (NPC, day 8), and kidney organoids (day over 21). *n* = 3 to 9. **(B)** qPCR of OAT1, OAT3, and OCT2 in undifferentiated ES cells, kidney organoids (day 21), and HKC8 cells. *n* = 3 to 9. *p < 0.05. **(C)** Gating of dissociated organoids by LTL. Even though only 22.60% of dissociated cells from organoids showed LTL positive, the mRNA level of OCT2 from organoids was comparable to that of HKC-8. **(D)** Localization of OAT1, OAT3, and OCT2 in kidney organoids (day 21). IF of podocalyxin (PODXL, green), LTL (cyan), OAT1 (red), OAT3 (red), and OCT2 (red). Podocytes did not express OAT1, OAT3, and OCT2 in kidney organoids. **(E–G)** Immunofluorescence (IF) of LTL (cyan), OAT1 (red), OAT3, and OCT2 in kidney organoids (day 51), human kidney samples, and HKC-8 cells. Scale bar: 50 μm.

To evaluate the expression of major drug transporters, we performed RNA-seq and qPCR analyses in human kidney organoids. Both RNA-seq and qPCR showed the expression of OAT1/3, OCT2, and PMAT in organoids (Figure 2A; Supplementary Figures S2A–D). qPCR revealed significantly higher expression of OAT1/3 in human kidney organoids than in HKC8, a human proximal tubular cell line (Figure 2B) (Schley et al., 2012). Although OCT2 expression levels were comparable between organoids and HKC-8, LTL+ proximal tubules accounted for only 22.60% in organoids as assessed by flow cytometry (Figure 2C), suggesting the mRNA level of OCT2 in organoids was 4-5 fold higher than that in HKC-8.

To confirm the protein expression of these key drug transporters, we performed immunostaining in organoids, human normal kidney samples, and HKC-8. Day 21 and 51 kidney organoids showed the expression of OAT1, OAT3, and OCT2 proteins on the epithelial cell membrane in LTL+ proximal tubules in contrast to PODXL+ podocytes, while day 10 and 14 organoids did not express these transporters (Figure 2D; Supplementary Figures S2E–G). SALL1, a renal progenitor gene, was detected in the early stages of organoids, yet its expression vanished after day 21, consistent with the maturation of organoids over time during culture (Supplementary Figures S2E–G) (Rizki-Safitri et al., 2022). Human kidney samples validated the immunostaining and the expression of OAT1, OAT3, and OCT2 in LTL+ proximal tubules, while HKC-8 showed weak OCT2 expression and a lack of OAT1 and OAT3. These results collectively suggest that organoid proximal tubules exhibit more physiological transporter expression than a traditional cell line, HKC-8.

### OAT-mediated drug uptake and injury in organoid proximal tubules

To evaluate whether kidney organoids recapitulate OAT-mediated drug uptake and injury in proximal tubules, we administered tenofovir and aristolochic acid (AA) which cause nephrotoxicity *via* OAT1-and OAT3-mediated transport from blood circulation into the cytoplasm of proximal tubular epithelial cells (Uwai et al., 2007; VanWert et al., 2010; Dickman et al., 2011; Kohler et al., 2011). Tenofovir is an acyclic nucleotide analogue reverse transcriptase inhibitor that is widely used for the treatment of human immunodeficiency virus type 1 (HIV-1) infection (Lyseng-Williamson et al., 2005). One major side effect is tubular dysfunction ranging from low-level proteinuria to full-blown Fanconi syndrome, associated with a progressive slow decline in renal function (Milburn et al., 2017). Although the mechanism remains unclear, the tenofovir toxicity is believed to be due to the mitochondrial stress and apoptosis of proximal tubule cells (Moss et al., 2014; Murphy et al., 2017). AA is a component of Chinese herbal medicine derived from *Aristolochia* plants which was originally identified as a contaminant of weight loss supplements. AA has been recognized as a nephrotoxic agent which causes progressive tubulointerstitial nephritis (Vanherweghem et al., 1993; Nortier et al., 2000; Hagos and Wolff, 2010). The mechanisms of cytotoxic effects appear to be defective activation of antioxidative enzymes, mitochondrial damage, DNA damage, impaired regeneration of proximal tubular epithelial cells (i.e., cell cycle arrest), endoplasmic reticulum and mitochondrial stress, activation of the caspase pathway and apoptosis (Vervaet et al., 2017).

**FIGURE 3:**
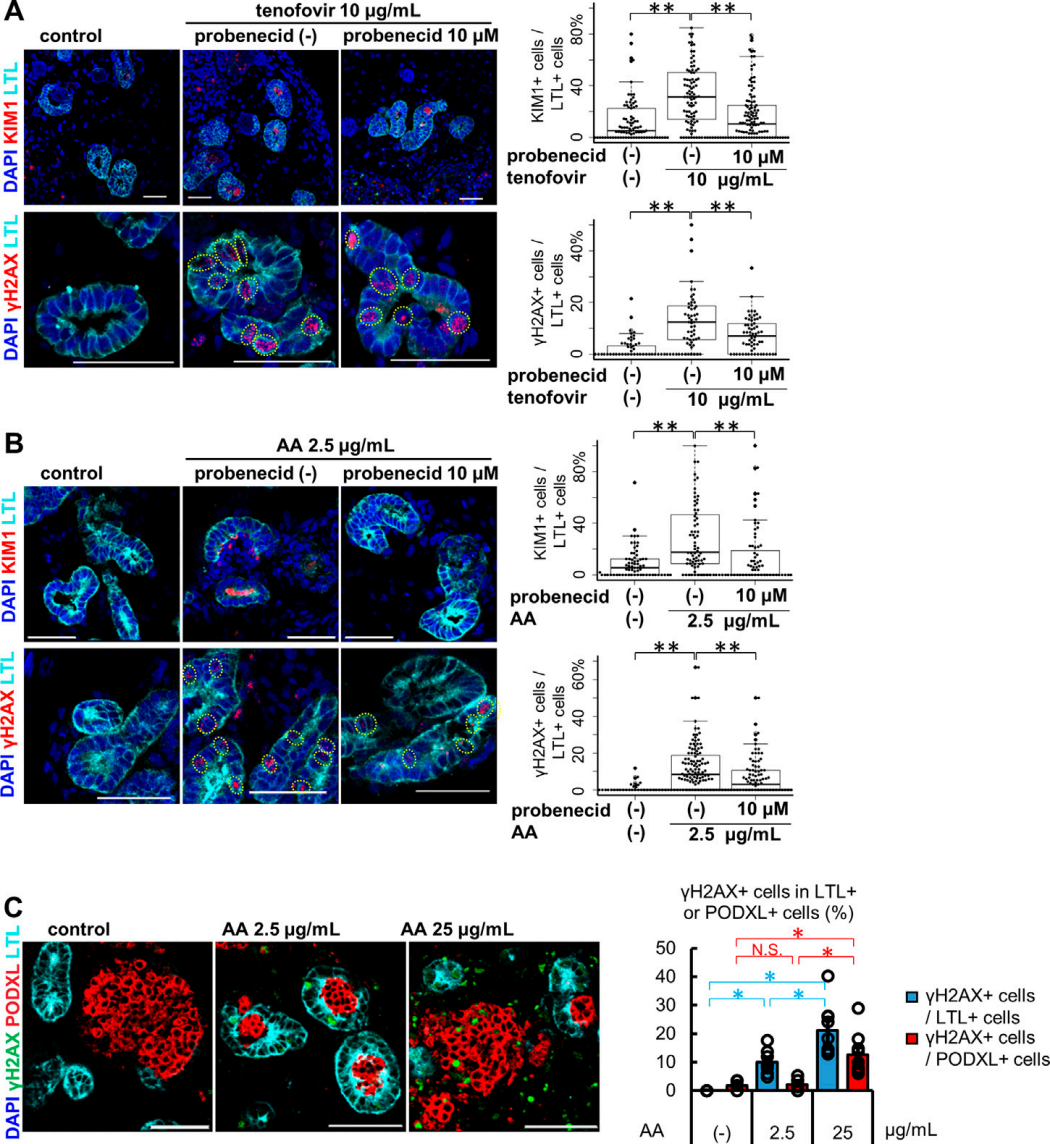
Tubule-specific injury in kidney organoids under the treatment of OAT1/3 mediated nephrotoxicants. **(A)** Representative IF images of KIM1 (red) and γH2AX (red) in the organoids (day53) treated with 10 μg/mL tenofovir for 7 days. Probenecid ameliorated the KIM1 and γH2AX increase caused by tenofovir. Experiments were repeated independently *n* = 3 times with similar results. **p < 0.01. Scale bar: 50 μm. **(B)** Representative IF images of KIM1 (red) and γH2AX (red) in the organoids (day 37) treated with 2.5 μg/mL AA for 24 h. Probenecid attenuated AA-induced proximal tubule injury. Experiments were repeated independently *n* = 3 times with similar results. **p < 0.01. Scale bar: 50 μm. **(C)** IF of γH2AX in the organoids treated with lower or higher doses (2.5 or 25 μg/mL) of AA. *p < 0.05. Scale bar: 50 μm.

Kidney organoids between differentiation days 37–59 were used for the toxicity assessment of OAT-mediated nephrotoxicants, as this was after peak OAT1/3 mRNA expression on day 21. After 7 days of treatment with 10 μg/mL tenofovir, kidney organoids exhibited tubular injury as reflected by KIM1 protein expression and DNA damage marked by γH2AX in LTL+ proximal tubules (Figure 3A). To assess whether the tenofovir-induced injury was mediated by OATs, we co-treated organoids with 10 μM probenecid, an OAT inhibitor (Uwai et al., 2007), during the tenofovir administration. Probenecid significantly reduced the percentage of KIM1+ proximal tubular cells from 31.3 [interquartile range, 13.8, 50.0] % to 10.3 [interquartile range, 0.0, 24.6] % and γH2AX+ proximal tubular cells from 12.6 [interquartile range, 6.2, 15.6] % to 7.1 [interquartile range, 0.0, 11.8] % after 1-week treatment with tenofovir (Figure 3A), suggesting that organoid tubules express functional OATs that can mediate tenofovir cellular uptake.

Next, we treated kidney organoids with 2.5 μg/mL AA for 24 h (Figure 3B). Both KIM1 and γH2AX were significantly increased after 24 h of AA treatment in LTL+ proximal tubules. As seen in tenofovir samples, probenecid rescued organoid tubules from AA-induced injury. The median positive ratio of KIM1+LTL+ tubular cells was significantly decreased from 17.7 [interquartile range, 8.7, 45.5] % to 0.0 [interquartile range, 0.0, 16.7] % of AA-treated organoids. Similarly, the median positive ratio of γH2AX was significantly decreased from 8.4 [0.0, 18.2] % to 3.0 [0.0, 10.5] %. These inhibitory effects of probenecid on AA toxicity were consistent with previous reports in animal models (Uwai et al., 2007; Bakhiya et al., 2009; Babu et al., 2010; Dickman et al., 2011; Xue et al., 2011; Baudoux et al., 2012). On the other hand, the induction of injury markers by 2.5 μg/mL AA was not observed in HKC-8. KIM1 was not detected by immunostaining in HKC-8 even with increased AA concentrations up to 25 μg/mL, while γH2AX positivity was increased at the higher concentration of 10 and 25 μg/mL (Supplementary Figure S3A). Given the absence of OAT1 and OAT3 expression in HKC-8 (Figures 2B–F), the injury response seen at higher concentrations of AA was thought to not be OAT-mediated but rather a non-specific mechanism that may not faithfully recapitulate the AA toxicity seen *in vivo*.

To address this question, kidney organoids were treated with 25 μg/mL AA for 24 h. Co-immunostaining for LTL and PODXL revealed the difference in segment-specificity of the injury response at different concentrations of AA (Figure 3C). LTL+ proximal tubules responded to both 2.5 and 25 μg/mL concentrations with γH2AX induction. Conversely, γH2AX was not increased in PODXL+ podocytes at 2.5 μg/mL, yet significant DNA damage was observed at 25 μg/mL in organoid podocytes. Together, these results suggest that kidney organoid tubules recapitulate OAT-mediated drug uptake and segment-specific injury responses more faithfully than traditional cell culture.

### OCT2-mediated proximal tubule injury in organoids

Cisplatin is widely used for solid tumor therapies of the head, neck, lung, testis, ovary, and breast cancers. The main side effect is OCT2-mediated nephrotoxicity which is observed in 30% of patients (Pabla and Dong, 2008). Cisplatin tends to accumulate in the proximal tubular cytoplasm due to higher uptake *via* OCT2 than efflux by MATE1, causing various cellular injury responses in proximal tubules (McSweeney et al., 2021). The major mechanism of cisplatin-induced nephrotoxicity is due to its direct binding to DNA, which leads to the arrest of DNA synthesis and replication (Wang and Lippard, 2005). In addition, cisplatin-induced renal toxicity involves multiple pathways including oxidative stress, activation of apoptotic cascades, and endonucleases (Miller et al., 2010).

**FIGURE 4:**
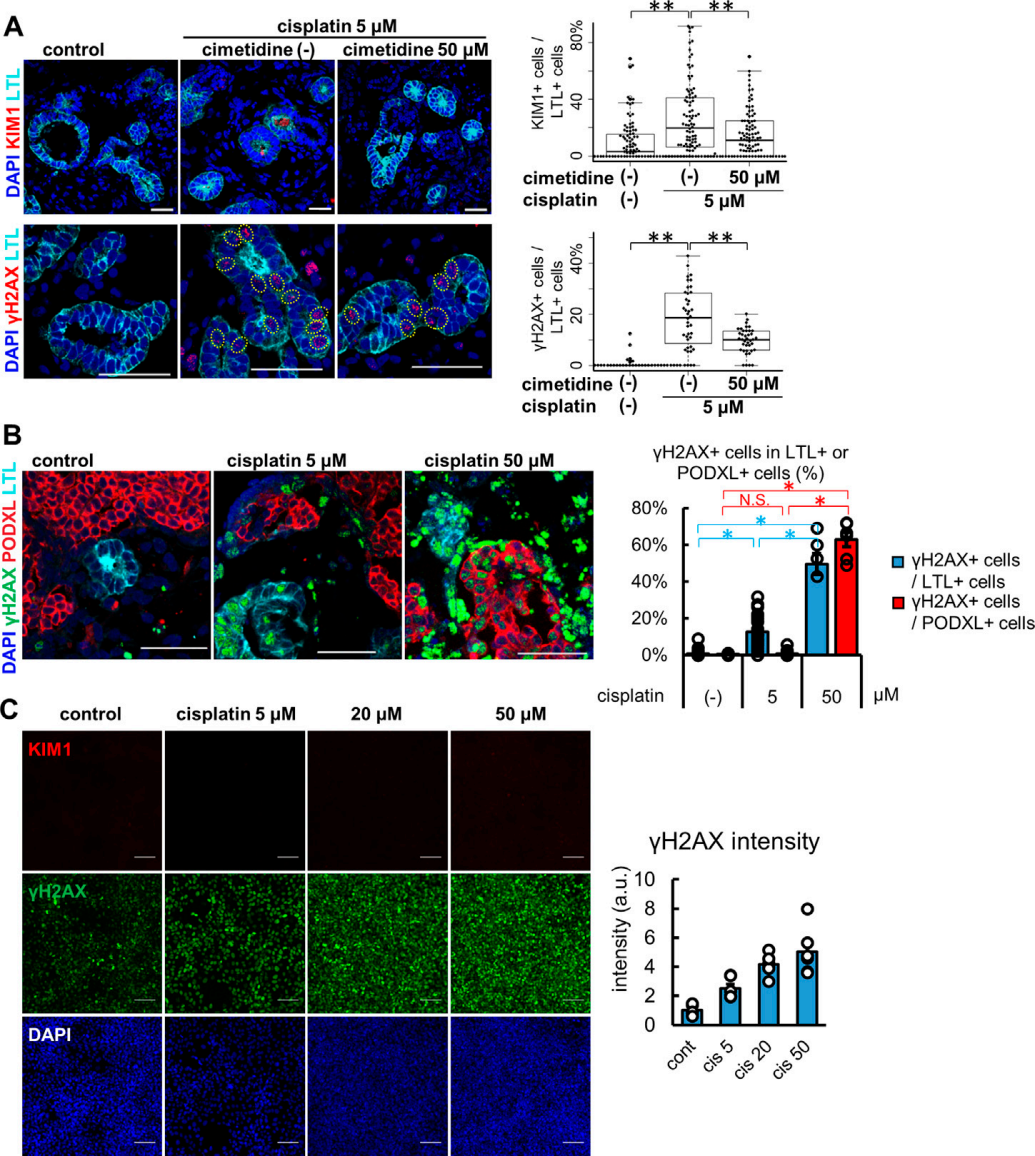
Injury responses to cisplatin treatment in kidney organoids and a proximal tubular cell line. **(A)** Representative IF images of KIM1 (red) and γH2AX (red) in the organoids treated with 5 μM cisplatin for 24 h. 50 μM cimetidine attenuated cisplatin-induced proximal tubule injury. Experiments were repeated independently *n* = 3 times with similar results. **p < 0.01. Scale bar: 50 μm. **(B)** IF of γH2AX in the organoids treated with lower or higher doses (5 or 50 μM) of cisplatin. *n* = 6. Scale bar: 50 μm *p < 0.05. **(C)** IF of γH2AX and KIM1 in HKC-8 cells treated with cisplatin and quantification of γH2AX intensity in arbitrary units (a.u.). *n* = 6. Scale bar: 100 μm.

Kidney organoids treated with 5 μM cisplatin for 24 h showed protein expression of KIM1 and γH2AX in LTL+ proximal tubules (Figure 4A). KIM1-positive tubular cells were significantly increased to 20.0 [6.6, 41.2] % from the control, 3.1 [0.0, 15.6] %. Co-treatment with 50 μM cimetidine, an OCT2 inhibitor reduced the KIM1-positivity down to 11.1 [0.0, 25.0] %. Likewise, compared with control (0.0 [0.0, 0.0] %), γH2AX-positive tubular cells were increased by cisplatin to 18.6 [9.3, 28.1] %, which were decreased to 10.0 [5.9, 13.3] % with cimetidine co-treatment. γH2AX-positive DNA damage was observed in LTL+ proximal tubules but not in PODXL+ podocytes with 5 μM cisplatin treatment, yet the high concentration of cisplatin at 50 μM resulted in widespread toxicity that induced DNA damage in podocytes (Figure 4B). Lastly, we compared the cisplatin-induced toxicity responses to a human proximal tubular cell line, HKC-8 at concentrations of 5, 20, and 50 μM (Figure 4C). γH2AX+ DNA damage was increased in a dose-dependent manner, consistent with the positive expression of OCT2 in HKC-8 (Figure 2B). However, KIM1 expression was not observed even at the highest concentration of 50 μM cisplatin in HKC-8 cells, highlighting the difference of kidney organoids as an improved model for toxicity assays *in vitro*.

### Kidney organoids for podocyte toxicity assessment

The podocyte is another important cell type for drug toxicity assessment. Puromycin aminonucleoside (PAN) is an aminonucleoside antibiotic known to cause podocyte injury and is widely used for experimental models of nephrotic syndrome in animals. PAN causes foot process effacement, actin cytoskeleton disorganization, and decreased expression and abnormal distribution of slit diaphragm proteins including nephrin and podocin (Mundel et al., 1997; Guan et al., 2004; Oh et al., 2004). PAN is transported into the cytoplasm of podocytes by PMAT (Xia et al., 2009). These toxic effects result in severe proteinuria and pathophysiological lesions resembling minimal change or focal segmental glomerular sclerosis in animal models (Hagiwara et al., 2006; Zheng et al., 2008).

**FIGURE 5:**
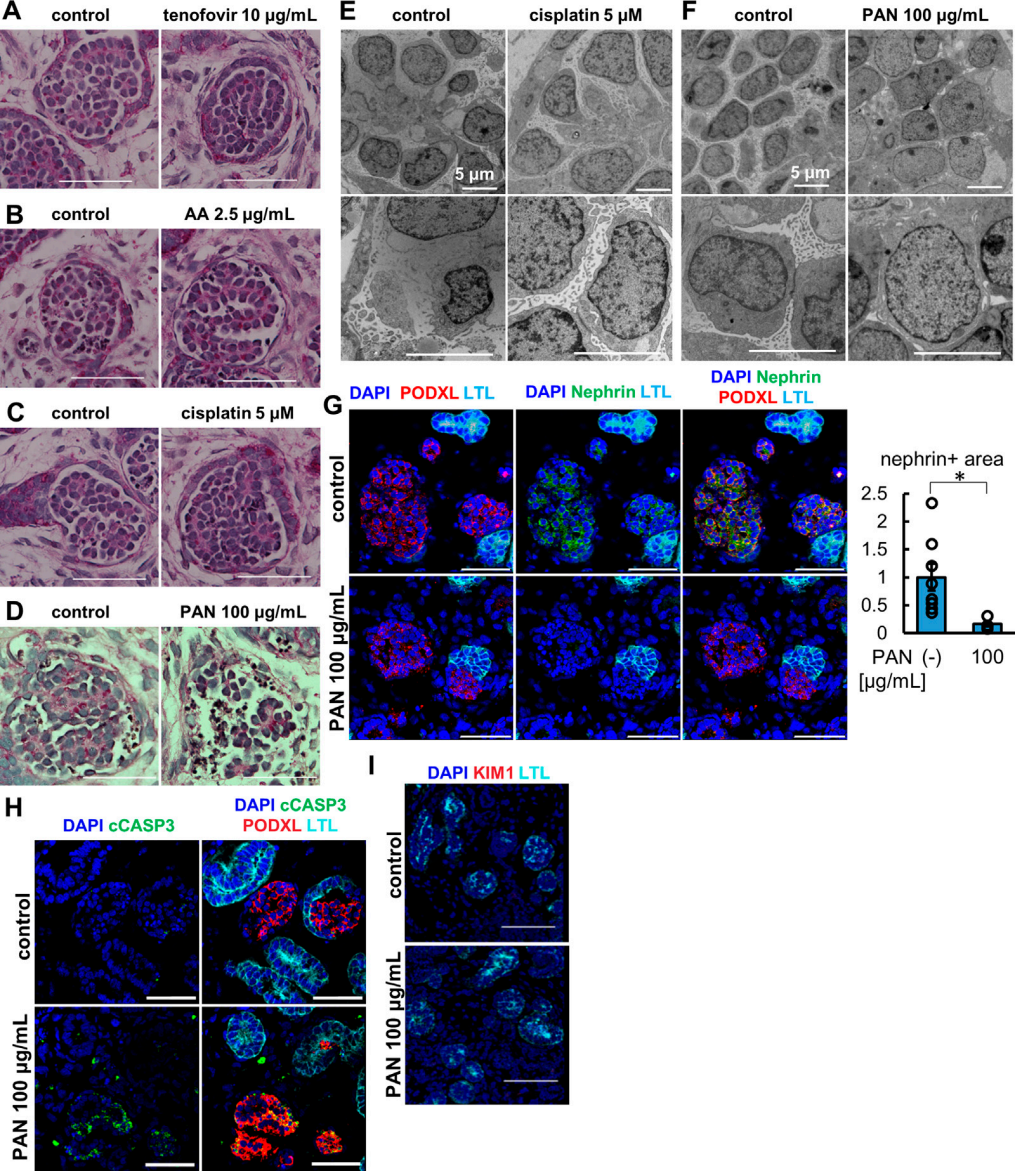
Glomerulus-specific injury in kidney organoids under the treatment of PAN. **(A–D)** PAS staining of glomerular structure in **(A)** the organoids treated with 10 μg/mL tenofovir for 7 days, **(B)** the organoids treated with 2.5 μg/mL AA for 48 h, **(C)** the organoids treated with 5 μM cisplatin for 24 h **(D)** the organoids treated with 100 μg/mL PAN for 24 h. Scale bar: 50 μm. **(E, F)** Electron microscopy images of glomerular structures in **(E)** the organoids treated with 5 μM cisplatin for 24 h and **(F)** the organoids treated with 100 μg/mL PAN for 24 h. Scale bar: 5 μm. **(G)** IF of nephrin (green), podocalyxin (PODXL, red), and LTL (cyan) in the organoids treated with 100 μg/mL PAN for 24 h. N = 8 glomerular structures from 3 independent organoids. *p < 0.05. Scale bar: 50 μm. **(H)** IF of cCASP3 (green), podocalyxin (PODXL, red), and LTL (cyan) in the organoids treated with 100 μg/mL PAN for 24 h. Scale bar: 50 μm. **(I)** IF of KIM1 (red) and LTL (cyan) in the organoids treated with 100 μg/mL PAN for 24 h. Scale bar: 50 μm.

We treated organoids with 100 μg/mL PAN for 24 h and analyzed the morphology of the glomeruli by light microscopy with comparison to the vehicle and tubular toxicant controls of tenofovir, AA, and cisplatin (Figures 5A–D). While the control samples including tubular toxicants did not show any apparent morphological change in glomerular podocytes, disruption of the podocyte structure was observed in PAN-treated organoids by PAS staining (Figure 5D). Transmission electron microscopy (TEM) revealed a substantial reduction of foot process-like structures in PAN samples compared to untreated controls (Figures 5E, F). To complement the TEM assessment on foot processes, we immunostained for nephrin, a slight diaphragm protein, that was significantly decreased in PAN samples compared with untreated controls (Figure 5G). This observation of nephrin loss was also confirmed by another podocyte toxicant, adriamycin (Supplementary Figure S4). Importantly, PAN treatment upregulated cleaved caspase 3 (cCASP3) expression in organoid podocytes, an indication of apoptosis (Figure 5H). Meanwhile, the lack of KIM1 induction in proximal tubules of PAN-treated organoids reflects segment-specific podocyte injury (Figure 5I). These results imply that kidney organoids can replicate glomerular toxicity responses and be used for podocyte toxicity assessment of drugs.

### Generation of ATP reporter organoids with a biosensor, perceval HR

**FIGURE 6:**
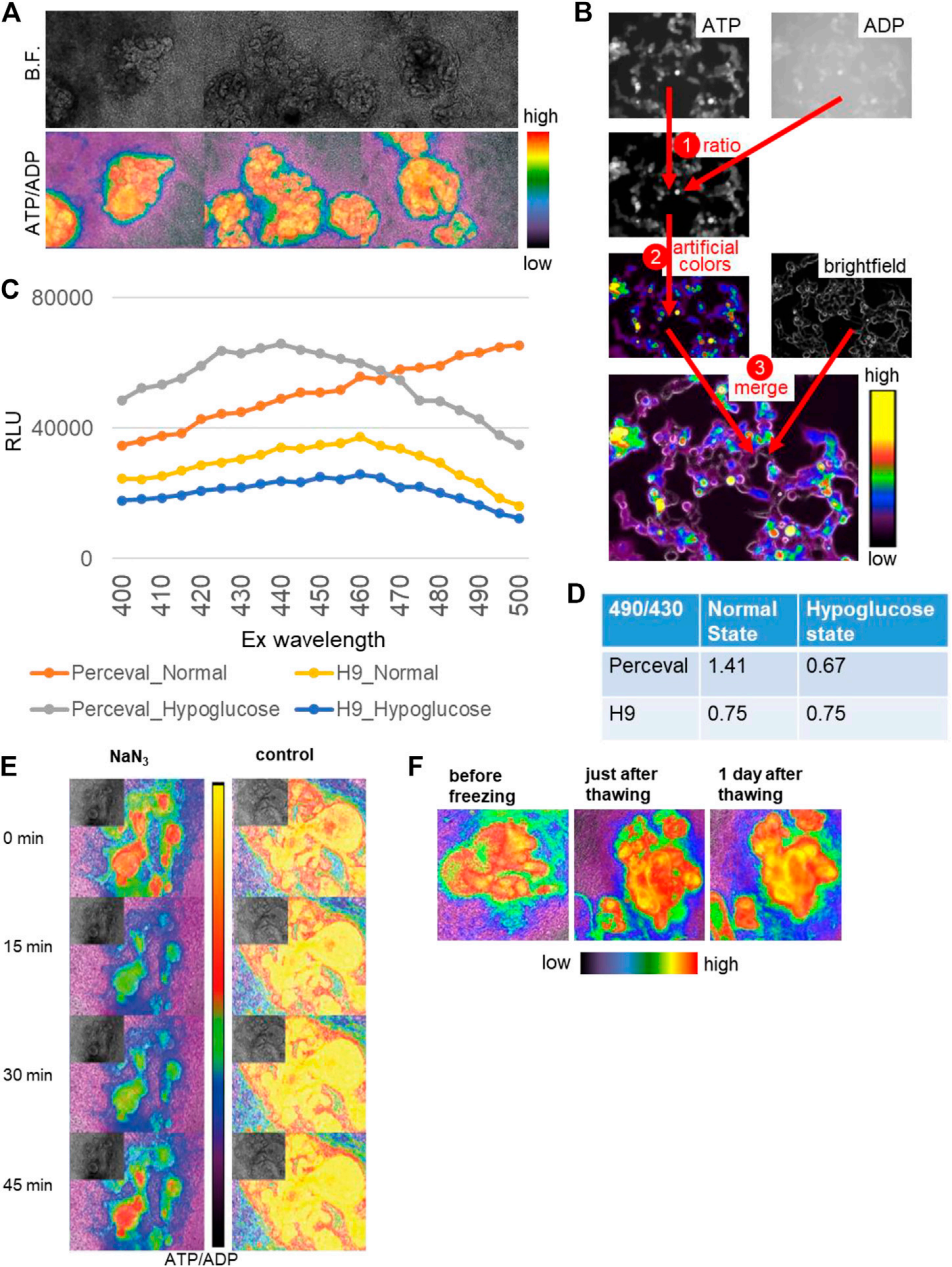
Reporter organoids with real-time biosensor Perceval HR to detect ATP/ADP ratio. **(A)** Bright-field (B.F.) and ATP/ADP ratio (lower) in nephron-like structures of 2D reporter organoids. **(B)** The channels of ATP (ex495/em540) and ADP (ex436/em540) are used for ratio calculation, transformed in artificial colors, and merged with the bright field. **(C)** Signal intensities at varied excitation wavelengths from 400 to 500 nm in the parental H9 organoids and Perceval H9 organoids in the normal and hypoglucose culture. **(D)** The signal ratios of em540 excited by 490 and 430 in the control and hypoglucose organoids, evaluated by a plate reader. The parental H9 was used as a control. **(E)** The reactivity of ATP/ADP ratio to toxic substance using NaN3. NaN3 was added at 0 min, and the same media were kept in the wells until 45 min. **(F)** 2D Reporter organoids before freezing, just after thawing, and 1 day after thawing.

During drug development, high-throughput screening is often used for therapeutic efficacy evaluation. Kidney organoids could be applied to high-throughput screening for renal toxicity assessment, yet the lack of reporter systems would make it difficult to increase the throughput. Therefore, we generated a reporter organoid model to evaluate the drug toxicity using Perceval HR, a genetically encoded fluorescent biomarker of ATP/ADP (Tantama et al., 2013). Since ATP serves as the principal regulator of cellular metabolism that drives many cellular reactions *via* converting into ADP, the ATP/ADP ratio can reflect cell health and vital cellular functions (Zanotelli et al., 2018). Perceval HR binds to both conformations of ATP and ADP and can be utilized to determine a concentration-independent ATP/ADP ratio as a metric of intracellular energy, and ATP and ADP levels can be quantified as signal intensities around 540 nm emission wavelength with 490 (ATP) and 430 (ADP) nm excitation wavelengths (Tantama et al., 2013). Hence, we generated stable hPSC lines expressing Perceval HR *via* lentiviral transduction and differentiated them into 2D and 3D kidney organoids as previously described (Figure 6A) (Morizane et al., 2015; Morizane and Bonventre, 2017a). ATP and ADP signals were acquired *via* live fluorescence microscopy, and the ATP/ADP ratios were computationally visualized based on the signal intensities of ATP and ADP (Figure 6B). The ATP/ADP ratios appeared to be higher in nephron-like structures than the surrounding stromal cells, consistent with high ATP production in epithelial cells (Liu et al., 2022) (Figure 6A).

To validate the Perceval HR system in organoids, we tested the biosensor organoids under hypoglycemic culture. We used varied excitation wavelengths ranging from 400 to 500 nm and quantified signal intensities at the 540 nm emission wavelength using a plate reader in whole kidney organoids derived from the parental H9 and Perceval HR lines (Figure 6C). The signal intensities excited at 490 and 430 nm were substantially higher in Perceval HR organoids than the controls. We then calculated the signal ratios of ATP and ADP excited at 490 and 430 nm respectively. While H9 controls did not show any change in the signal ratios, Perceval HR organoids decreased the ATP/ADP ratios to 0.67 under hypoglycemic culture from 1.41 in the normal culture media (Figure 6D). Next, we tested a toxic substance, namely, sodium azide (NaN3), a cytochrome oxidase inhibitor which is used for chemical hypoxic experiments. 5 mM NaN3 induced a rapid decrease of the ATP/ADP ratio in nephron structures within a minute, followed by spontaneous recovery in 45 min (Figure 6E). Lastly, we examined whether these Perceval HR organoids can be stored as frozen stocks that can be practical for future experiments. The organoids were frozen in differentiation media supplemented with 10% DMSO and stored at −80°C. After a few weeks, the organoids were thawed and recovered with an ATP/ADP ratio similar to pre-freezing conditions (Figure 6F).

### Nephrotoxicity assessment in perceval HR organoids

**FIGURE 7:**
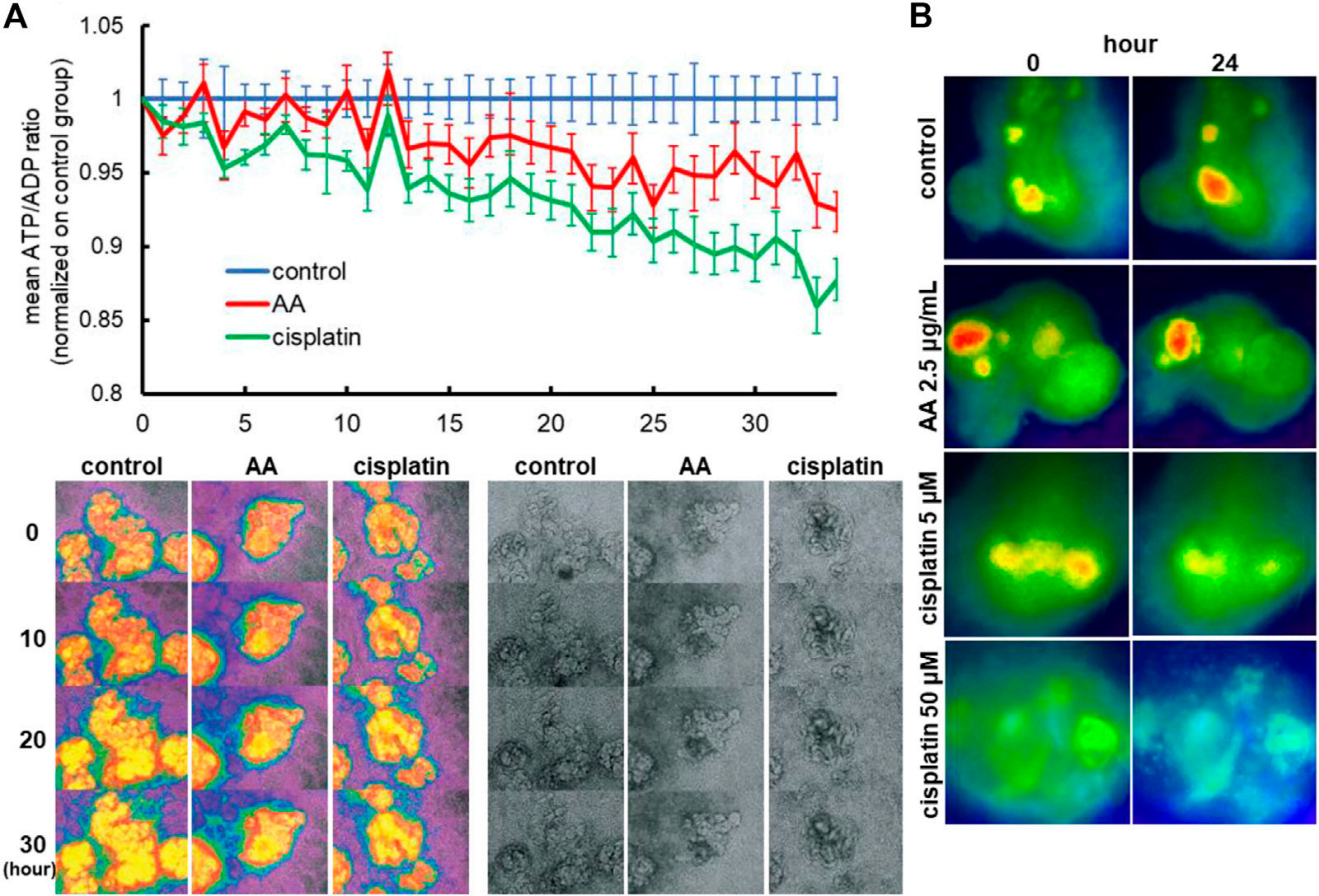
Reporter organoids with real-time biosensor Perceval HR to detect ATP/ADP ratio. **(A)** Detection of nephrotoxicity by live monitoring of ATP/ADP ratio in 2D reporter organoids treated with 2.5 μg/mL AA and 5 µM cisplatin. **(B)** Representative images of ATP/ADP ratio monitoring in minimized 3D reporter organoids cultured on 384-well plates. The organoids were exposed to various toxicants (2.5 μg/mL AA, 5 or 50 µM cisplatin).

To evaluate whether the ATP/ADP ratios can be used as a readout of nephrotoxicity assays, we treated Perceval HR organoids with 2.5 μg/mL AA or 5 µM cisplatin and monitored ATP and ADP signals by time-lapse imaging in 2D organoids. AA and cisplatin reduced ATP/ADP ratios over time during 2-day imaging as compared to the control (Figure 7A). Similarly, the reduction of ATP/ADP ratios by nephrotoxicants was observed in 3D kidney organoids generated in 384-well plates that are commonly used for high-throughput screening (Figure 7B).

**FIGURE 8:**
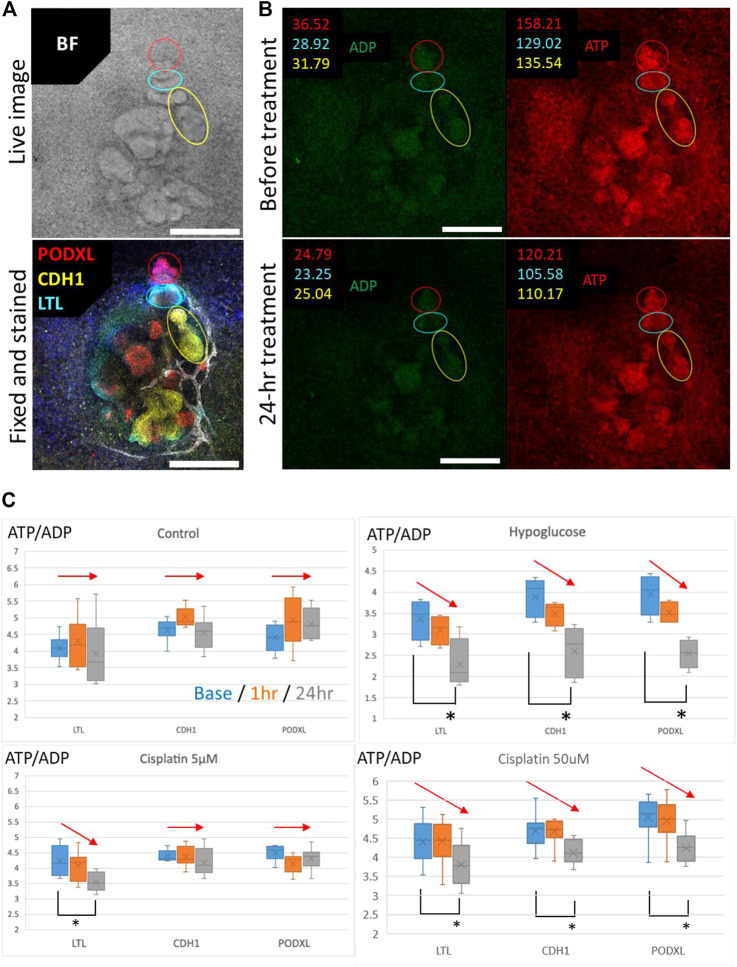
Evaluation of the segment-specific injury by Perceval HR organoids. **(A)** Representative BF image and IF image of PODXL (red), LTL (cyan), and CDH1 (yellow) in 2D organoids. Scale bars: 200 μm. **(B)** Representative images for quantification of ATP/ADP ratio matched in position with the BF and IF images for segment identification in 2D organoids exposed to 5 μM cisplatin. Scale bars: 200 μm. **(C)** Detection of ATP/ADP ratio of each segment in 2D reporter organoids treated with hypoglucose, 5 and 50 μM cisplatin for 24 h *n* = 4 to 15. *p < 0.05.

To validate whether segment-specific injury can be evaluated by Perceval HR organoids, we fixed the organoids after time-lapse imaging of ATP/ADP signals and stained for PODXL (podocytes), LTL (proximal tubules), and CDH1 (loops of Henle and distal nephrons) (Figure 8A). The ATP and ADP signal intensities were then quantified in the lesions of PODXL^+^, LTL^+^, and CDH1^+^ cells in the time-lapse images (Figure 8B). The ATP/ADP ratios remained same in all 3 cell types in the control samples during 24-h imaging, while hypoglycemic culture significantly reduced the ATP/ADP ratios in all cell types after 24 h. 5 μM cisplatin reduced the ATP/ADP ratios in only LTL+ tubules but not in CDH1+ nor PODXL+ cells. In contrast, the high concentration of cisplatin at 50 μM significantly decreased the ATP/ADP ratios in all cell types (Figure 8C). These results are consistent with the nephrotoxic evaluation by immunostaining for KIM1 and γH2AX, suggesting that the ATP/ADP biosensor can be used for segment-specific toxicity assessment in live kidney organoids.

## Discussion

Drug-induced kidney injury is a serious problem in clinical settings and a major barrier to drug discovery. Hence, effective and accurate prediction of nephrotoxicity is extremely important. In this study, we investigate the applicability of human pluripotent stem cell-derived kidney organoids as a novel platform for drug toxicity assessment *in vitro*. We confirm that kidney organoids express drug transporters, OAT1, OAT3, and OCT2, in a segment-specific manner, while HKC-8, a human proximal tubule cell line, does not express OAT1 and OAT3. This difference can explain why kidney organoids exhibit a more sensitive injury response to AA than HKC-8. Another important difference is that KIM-1 is not detected in HKC-8 even with AA treatment at high concentrations, whereas LTL+ proximal tubules in organoids express KIM1 on apical membranes after treatment with various tubular toxicants including AA, cisplatin, and tenofovir.

In kidney organoids, tenofovir- and AA-induced injury was ameliorated by an OAT inhibitor, probenecid, suggesting organoids express functional OATs that mediate cellular uptake of those drugs. Similarly, the cisplatin-induced injury was rescued by an OCT2 inhibitor, cimetidine. However, cimetidine did not completely prevent DNA damage and KIM1 upregulation. One possible reason for the partial rescue by cimetidine is that cisplatin uptake into proximal tubule cytoplasm is mediated by multiple transporters in addition to OCT2. Several *in vitro* and *in vivo* studies verified that cisplatin uptake into the cytoplasm is also handled by copper transporter 1 (CTR1) and megalin in human proximal tubules (Camano et al., 2010; Ciarimboli, 2014; Hori et al., 2017). Another explanation may reside in the inhibitory action of cimetidine on the apically expressed multidrug extrusion transporter-1 (MATE1) (Ito et al., 2012), which mediates cisplatin efflux into lumens. Indeed, a recent study has shown the longitudinal maturation of drug efflux function *via* multidrug resistance 1 (MDR1) in human kidney organoids by live imaging using Rhodamine (Rizki-Safitri et al., 2022). Of note, rodent studies reported that the protective effects of cimetidine on cisplatin-induced nephrotoxicity were incomplete (Ciarimboli et al., 2010), consistent with our results in kidney organoids. Hence, kidney organoids appear to replicate drug-induced AKI which is mediated by functional drug transporters in proximal tubular cells, thereby overcoming disadvantages of current *in vitro* and *in vivo* tools for nephrotoxicity assessment.

Another important advantage of kidney organoids may be the capability to assess drug toxicities to other nephron segments in addition to proximal tubules. The tubular toxicants of tenofovir, AA, and cisplatin induced proximal tubular injury at low concentrations, while there was no apparent damage to podocytes. In contrast, PAN and adriamycin induced loss of nephrin and foot processes in organoid podocytes, consistent with previous studies in animals. This feature of multicellular kidney organoids is potentially useful to distinguish physiological toxicity responses from general toxicity that can be induced by supraphysiologic concentrations of drugs.

One future direction of organoid research may be high-throughput drug screening. To this end, we generated a reporter line that can detect ATP and ADP production using a biosensor, Perceval HR, in live organoids. Our results suggest that the reduction in ATP/ADP ratios is an indicator of drug toxicity in kidney organoids, offering a direct live readout in high-throughput formats. Compared to time-consuming conventional methods of immunostaining, immunoblotting, and qPCR, the live monitoring of ATP/ADP expression may enable high-throughput screening for drug toxicity assessment in organoids. Interestingly, the ATP/ADP expression in organoids was stable even after the freeze-thaw cycle; therefore, large numbers of organoids can be prepared and stored before the high-throughput screening.

One limitation of this present study is that the efflux function was not evaluated other than by transcriptomics. According to a recent study of live functional assessment on drug secretion function, an apical transporter, MDR1, is not highly expressed in young organoids on day 21 of differentiation, while OCT2 is already expressed at the early stage (Rizki-Safitri et al., 2022). Hence, the injury response could be stronger in young organoids than in older organoids that can remove drugs from the tubular cytoplasm by efflux. To address this issue, we used only older organoids between days 37 and 59 for nephrotoxicity assays. Hence, it is recommended to evaluate both apical and basal transporters when drug toxicities are assessed in human kidney organoids.

To summarize, we assessed the capability of hPSCs-derived kidney organoids for drug toxicity testing. Kidney organoids mimic drug-induced tubular and glomerular injury in a segment-specific manner, providing a new tool for nephrotoxicity assays. ATP/ADP biosensor organoids may be useful for toxicity assessment in a high-throughput manner.

## Methods

### Generation of human induced pluripotent stem cells from blood T lymphocytes

One iPS cell line named TCiPS1 was generated using Sendai virus-mediated introduction of Yamanaka reprogramming factors (Yamanaka, 2007). T lymphocytes were isolated from peripheral whole blood using BD Vacutainer CPT Tube (BD Biosciences). Isolated T lymphocytes were then expanded on tissue culture plates coated with anti-CD3 antibody. Reprogramming was performed by Sendai virus vectors with OCT3/4, SOX2, KLF4, and c-MYC (CytoTune-iPS 2.0, Dnavec, Tsukuba, Japan). The multiplicity of infection was set to 5. 24 hours after infection, T lymphocytes were seeded on Geltrex-coated culture plates. Approximately 20 days later, 6 ES cell-like colonies were selected. For the confirmation of the characteristics of typical iPS cells, we examined the pluripotency and differentiation potential by the expression of pluripotency markers and embryoid body formation, respectively. Immunostaining revealed that these clones expressed pluripotency markers, TRA1-60, SOX2, NANOG, and OCT3/4 (Supplementary Figure S1B). The differentiation capability into three germ layers (GATA4 as an endoderm marker, FOXF1 as a mesoderm marker, and β III tubulin as an ectoderm marker) was observed in embryoid bodies of day 14, which was developed from iPS cells by cultivating in differentiation media supplemented 2% fetal bovine serum (Supplementary Figures S1C, D). The clone was confirmed as karyotypically normal (46, XY) after more than twenty culture passages (Supplementary Figure S1E). To confirm line identity, short tandem repeat (STR) analysis was performed using genomic DNA extracted from T lymphocytes from the healthy volunteer and TCiPS1 cells (Supplementary Figure S1F). Routinely media samples were tested for the absence of *mycoplasma* contaminations by MycoAlertTM PLUS *Mycoplasma* Detection Kit (Lonza). The clearance of the vectors and the exogenous reprogramming factor genes were confirmed by qPCR after thirty-five culture passages.

### Maintenance of hPSCs

H9 (WiCell) human embryonic stem cells and TCiPS1 induced pluripotent stem cells were maintained on hESC-qualified Geltrex (Thermo Fisher Scientific) coated plates in feeder-free culture using StemFit ^®^ Basic02 (Ajinomoto Co., Inc.) supplemented with 10 ng/mL of FGF2 (Peprotech), as previously reported (Morizane and Bonventre, 2017a). The hPSC line was passaged weekly, using Accutase (STEMCELL technologies) for dissociation and Y27632 (Tocris) to facilitate adhesion on passaging. H9 passage numbers 50–62 and TCiPS1 passage numbers 18–30 were used for all experiments.

### Differentiation of hPSCs and 3D kidney organoid formation

The directed differentiation of hPSCs into kidney organoids is covered in detail elsewhere (Morizane and Bonventre, 2017a). Briefly, hPSCs were differentiated into metanephric mesenchyme cells which include nephron progenitor cells, with approximately 80%–90% efficiency, by a 3-step directed differentiation protocol. Metanephric mesenchyme cells, arising on day 8 of differentiation, were transferred into suspension culture in 96- or 384-well ultra-low adhesion plates (Fisher Scientific) and further differentiated into kidney organoids through intermediate stages of pretubular aggregates (day 11) and renal vesicles (day 14) as previously reported.

### Maintenance of 2D kidney organoid

Kidney organoids were maintained on 24-well plates in 500 μL of Advanced RPMI (ARPMI, Fisher Scientific) supplemented with 1% (v/v) GlutaMAX (Fisher Scientific) following induction of renal vesicles at day 14 of differentiation. Media changes were conducted three times weekly.

### Maintenance of 3D kidney organoid

Kidney organoids were maintained on 96- or 384-well plates in 200 μL or 80 μL of Advanced RPMI (ARPMI, Fisher Scientific) supplemented with 1% (v/v) GlutaMAX (Fisher Scientific) following induction of renal vesicles at day 14 of differentiation. Media changes were conducted three times per week.

### Nephrotoxicants treatment

3D kidney organoids differentiated from H9 or TCiPS1 cells were cultured in a basic differentiation medium supplemented with 10 μg/mL tenofovir (TOCRIS, #3666), 2.5 μg/mL AA (Sigma, #A9451), 5, 20 and 50 μM cisplatin (Sigma, #P4394), 100 μg/mL PAN (Cayman Chemical, #15509), and 10 μM adriamycin (Cayman Chemical, #15007) for 24, 48 h or 7 days after day 23 to 59 of differentiation. For inhibition of OAT1/3 or OCT2, 10 μM probenecid (Sigma, #P8761) or 50 μM cimetidine (Sigma, #C4522) were also supplemented. After the treatment, organoids were harvested for immunostaining with 4% paraformaldehyde (Electron Microscopy Sciences, #RT15710) or for qPCR with TRIzol.

### Immunocytochemistry

Cells were fixed with paraformaldehyde (4%) in PBS for 1 h. Immunofluorescence was performed as previously described (Morizane et al., 2015). The primary antibodies used were anti- TRA1-60 (Millipore, #MAB-4360), anti-SOX2 (Santa Cruz, #SC-17320), anti-SALLI (R&D systems, PP-K9814-00), anti-OCT3/4 (Santa Cruz, #SC-5279), anti-NANOG (ReproCell, #AB001P), anti-OAT1 (biorbyt, #orb11177), anti-OAT3 (NOVUS, #NBP1-92396), anti-OCT2 (NSJ, R31806), anti-KIM1 (R&D systems, AF1750), and anti-LTL (Vector lab, B-1325). Alexa 488, 555, or 647 dye-labeled (Molecular Probes; Invitrogen) secondary antibodies were used for immunofluorescence. Immunofluorescence images were obtained using C1 confocal (Nikon) or Stellaris 8 (Leica).

### Immunohistochemistry of embryoid bodies and 3D organoids

Embryoid bodies or kidney organoids were fixed with paraformaldehyde (4%) in PBS for 1 h. Immunofluorescence was performed as previously described (Morizane et al., 2015). The primary antibodies used were anti-GATA4 (Santa Cruz, #SC-1237), anti-FOXF1 (R&D systems, #AF4798), anti-β III tubulin (Millipore, #MAB1637), anti-OAT1, anti-OAT3, anti-OCT2, anti-KIM1, anti-γH2AX (Cell Signaling, #2577), anti-nephrin (Progen Biotechnik, GP-N2), anti-LTL, anti-podocalyxin (R&D systems, #1658), and Phalloidin (Molecular Probes, #R-415). Alexa 488, 555, or 647 dye-labeled (Molecular Probes; Invitrogen) secondary antibodies were used for immunofluorescence. For 3D whole-mount immunohistochemistry, kidney organoids underwent an additional clearing process after immunostaining as previously described (Hiratsuka et al., 2019). Immunofluorescence images were obtained using C1 confocal (Nikon) or Stellaris 8 (Leica).

### Quantitative RT-PCR

Quantitative PCR analysis was performed on the kidney as previously described (Morizane et al., 2015). Total RNA from human kidney organoids was extracted using TRIzol reagent (Invitrogen, Carlsbad, CA, USA), according to the manufacturer’s instructions. Total RNA was reverse transcribed using a High-Capacity cDNA Reverse Transcription Kit (Life Technologies, #4368814). Quantitative real-time PCR by iQ5 Multicolor Real-Time PCR Detection System (Bio-Rad) was performed using the primer sets shown as follows:

OAT1: CTG​GTT​CTT​CAT​TGA​GTC​GGC/GCC​CGG​AGT​ACC​TCC​ATA​C.

OAT3: TCG​ATG​TCC​CAG​CCA​AGT​TC/TCA​CGG​TCT​GCA​AGT​CCA​AG.

OCT2: CTC​CCC​AAG​ACA​GTG​TAG​GC/TAC​CAG​GTT​AAA​CTC​GGT​GAC​G.

KIM1: CTT​ACA​CAA​CAG​ATG​GGA​ATG​AC/TGG​CCG​TCA​GTA​GAC​TAT​GTT​C.

PMAT: GCC​TAC​ATG​CGC​TTT​GAT​GT/CAG​TAA​CAG​GGC​TCT​GAA​GGT​G.

L-FABP: CAG​GGG​AGA​AAG​TCA​AGA​CAG​T/CGT​TGA​GTT​CGG​TCA​CAG​AC.

Sendai virus genome: GGA​TCA​CTA​GGT​GAT​ATC​GAG​C/ACC​AGA​CAA​GAG​TTT​AAG​AGA​TAT​GTA​TC.

OCT3/4: CCC​GAA​AGA​GAA​AGC​GAA​CCA​G/AAT​GTA​TCG​AAG​GTG​CTC​AA.

SOX2: ACA​AGA​GAA​AAA​ACA​TGT​ATG​G/ATG​CGC​TGG​TTC​ACG​CCC​GCG​CCC​AGG.

KLF4: ACA​AGA​GAA​AAA​ACA​TGT​ATG​G/CGC​GCT​GGC​AGG​GCC​GCT​GCT​CGA​C.

C-MYC: TAA​CTG​ACT​AGC​AGG​CTT​GTC​G/TCC​ACA​TAC​AGT​CCT​GGA​TGA​TGA​TG.

### Transmission electron microscopy analysis

For morphological analysis, 3D kidney organoids were fixed with 4% PFA for 20 min and subsequently fixed with EM fixation buffer consisting of 1.5% glutaraldehyde, 1% paraformaldehyde, 70 mM NaPO4 pH 7.2, and 3% sucrose in water overnight at 4°C. The organoids were washed three times in 0.2 M cacodylate buffer pH 7.4 for 10 min each and fixed in a solution of 1% OsO4 for 1 h on ice. The organoids were then washed three times in 0.2M sodium cacodylate buffer pH 7.4 for 10 min each, dehydrated in a series of 70, 80, 90, and 100% ethanol. Samples were infiltrated with a mixture of 1:1 Epon LX-112 and propylene oxide for 1 h, followed by infiltration with pure Epon for 2 h. Subsequently, the samples were embedded in pure Epon, mounted in Beem capsules, and polymerized for 48 h at 60°C. 70 nm sections were cut and collected onto copper slot grids covered with formvar film and a 7 nm carbon layer and stained with an aqueous solution of 7% uranyl acetate for 20 min, followed by Reynold’s lead citrate for 10 min. All imaging was performed at 80.0 kV on JEOL JEM1400 and captured with AMT Image Capture Engine software.

### Live-cell fluorescence microscopy for imaging changes in ATP/ADP

Lentivirus carrying Perceval HR was produced in HEK293 cells by transfection of FUGW-Perceval HR (addgene) (Tantama et al., 2013) and was transduced to H9. ATP and ADP signals were acquired *via* live fluorescence microscopy, and a ratio was quantified with the ImageJ RatioPlus plugin.

## Statistics

Data are presented as means ± SE. A Student’s *t*-test was used for comparisons between groups. ANOVA and Tukey’s test was used for multiple comparisons.

## Data Availability

Publicly available datasets were analyzed in this study. This data can be found here: https://ddbj.nig.ac.jp/public/ddbj_database/dra/fastq/DRA010/DRA010266/.
