# Supplementary material for: ATP/ADP biosensor organoids for drug nephrotoxicity assessment
Source: Front Cell Dev Biol. 2023 Mar 2;11:1138504. doi: 10.3389/fcell.2023.1138504 (PMC10017499; doi:10.3389/fcell.2023.1138504)
Supplement: Supplementary file 1 [file DataSheet1.docx]

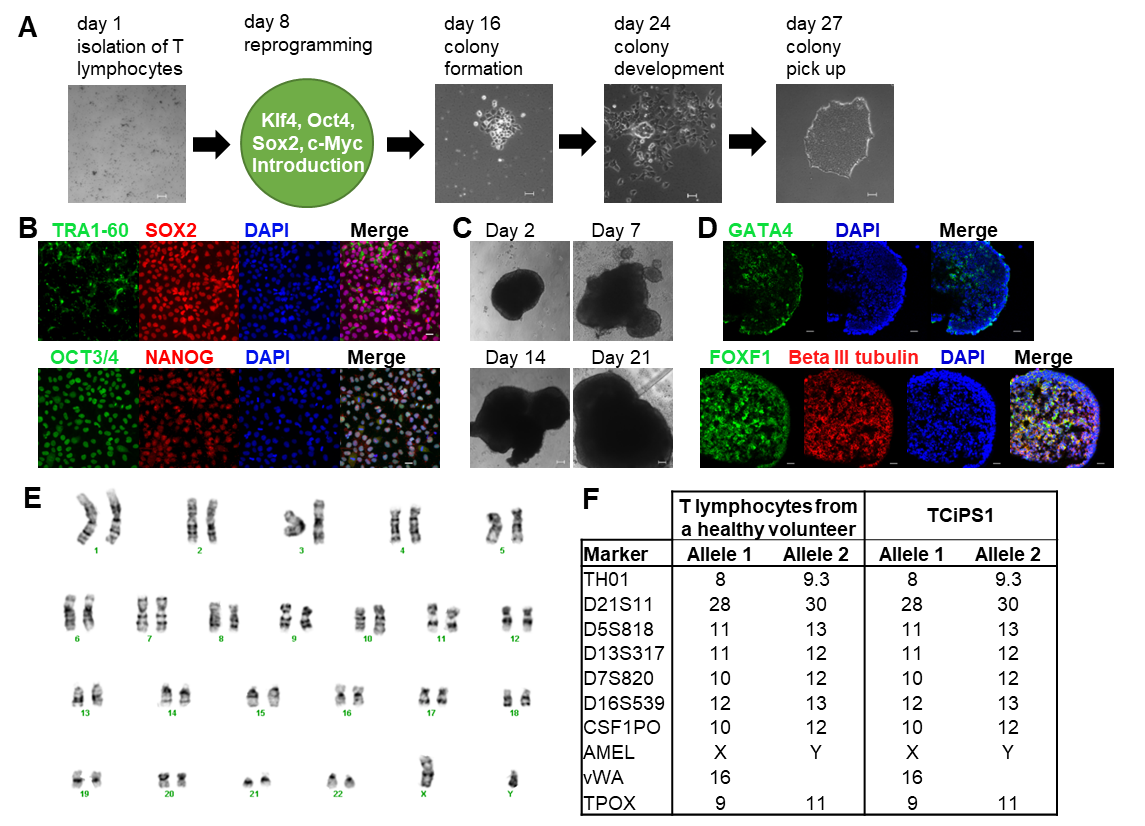


**Fig. S1. Generation and characterization of human induced pluripotent stem cells from blood T lymphocytes.**

(A) Representative steps of the generation of iPS cells (named TCiPS1) from isolated T lymphocytes. Scale bar: 50 μm.

(B) IF of pluripotency markers in undifferentiated TCiPS1 cells. All TRA1-60, SOX2, OCT3/4, and NANOG were abundantly expressed. Scale bar: 50 μm.

(C) Bright-field images of stochastically differentiated TCiPS1 cells through embryoid body formation. Scale bar: 50 μm.

(D) IF of GATA4 (an endoderm marker), FOXF1 (a mesoderm marker), and β III tubulin (an ectoderm marker) in the embryoid body of day 14 differentiated from TCiPS1 cells. Scale bar: 50 μm.

(E) Giemsa banding karyotype analysis of TCiPS1 cells. TCiPS1 cells reveal a normal karyotype.

(F) STR analysis of T lymphocytes from a healthy volunteer and TCiPS1 cells. All of the 10 analyzed loci matched between the two DNA profiles.


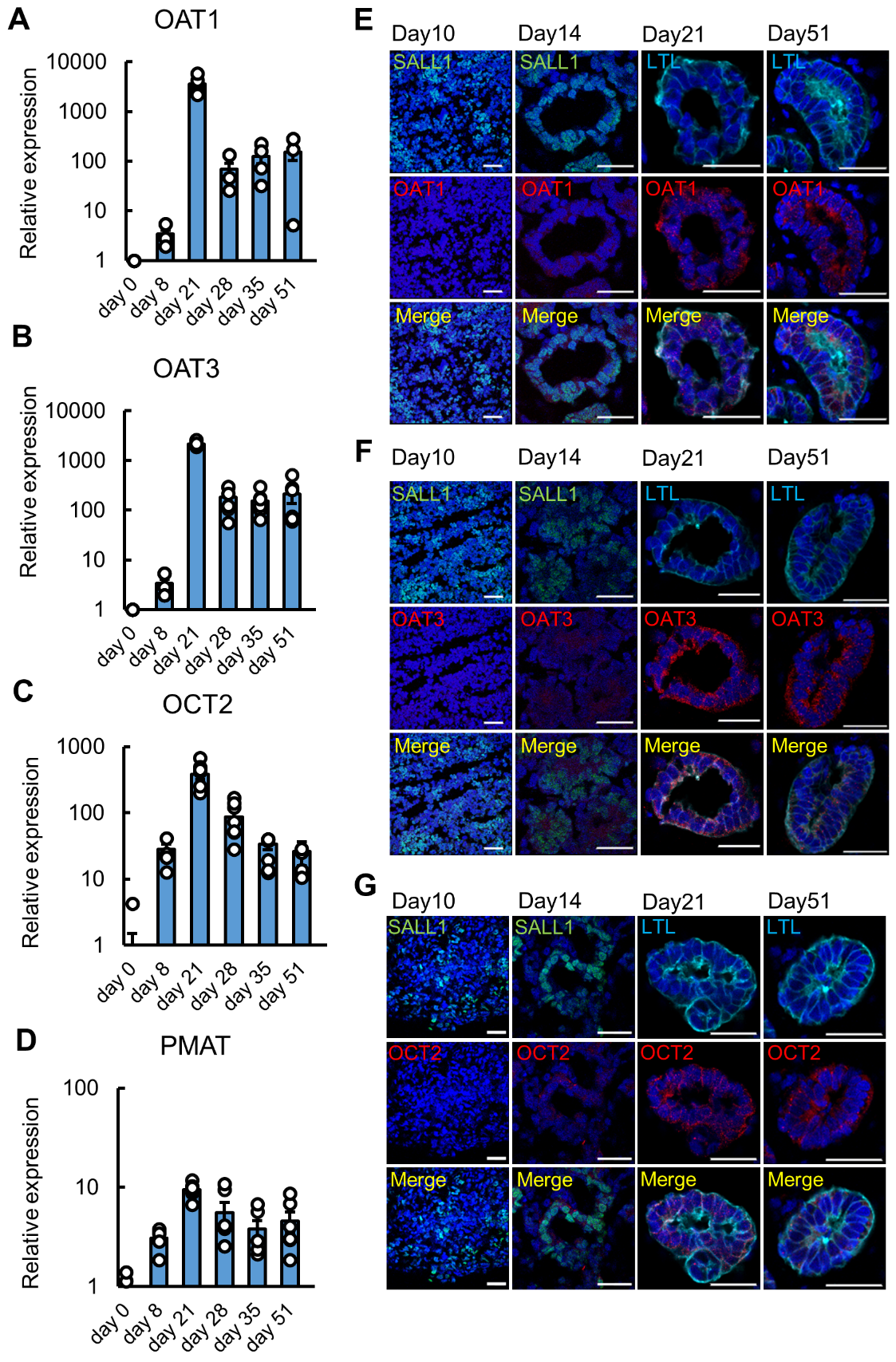


**Fig. S2. Expression of OAT1/3, OCT2, and PMAT in various differentiation steps of kidney organoids.**

(A, B, C) Quantitative real-time PCR of OAT1/3 and OCT2 in undifferentiated ES cells (day 0), metanephric mesenchyme (day 8), and kidney organoids (day 21, 28, 35, and 51). n = 3 to 9.

(D) Quantitative real-time PCR of PMAT in undifferentiated ES cells (day 0), metanephric mesenchyme (day 8), and kidney organoids (day 21, 28, 35, and 51). n = 9.

(E, F, G) Immunofluorescence (IF) of SALL1 (green), LTL (cyan), OAT1 (red), OAT3 (red), and OCT2 (red) in day 10, 14, 21, and 51 of kidney organoids. Scale bar: 50 μm.


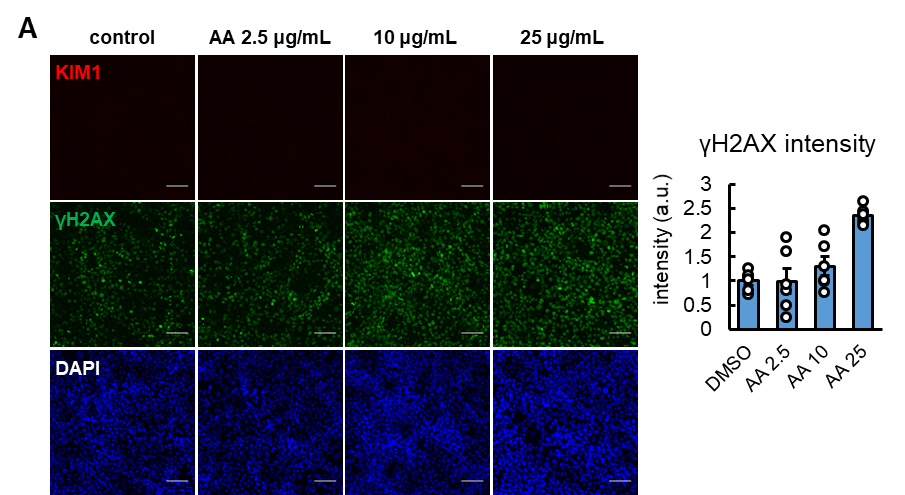


**Fig. S3. Injury of HKC-8 cells under the treatment of AA.** (A) IF of γH2AX and KIM1 in HKC-8 cells treated with AA and quantification of γH2AX intensity in arbitrary units (a.u.). n = 6. Scale bar: 100 μm.


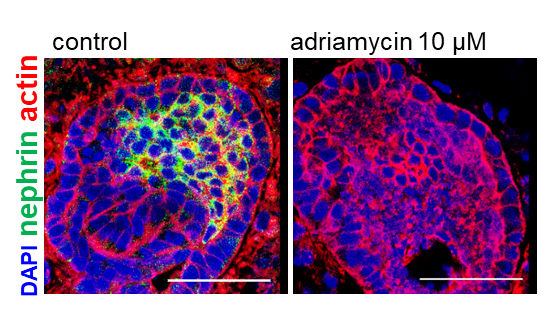


**Fig. S4. Glomerulus-specific injury in kidney organoids under the treatment of adriamycin.**

IF of nephrin (green) and actin (red) in the organoids treated with 10 μM adriamycin for 24 hours. Adriamycin induced a decrease in nephrin expression and disruption of actin organization in glomerulus-like structures of kidney organoids. Scale bar: 50 μm.
